# Silent Giant: Surgical Resection of a Large Biliary Cystadenoma in a 47-Year-Old Male With Underlying Steatohepatitis

**DOI:** 10.7759/cureus.105763

**Published:** 2026-03-24

**Authors:** Maitha Ibrahim Al Hammadi, Asia Abdalla S Mohamed Abdalla, Mark W Noble, Rakesh Rai

**Affiliations:** 1 College of Medicine, University of Sharjah, Dubai, ARE; 2 College of Medicine, University of Sharjah, Sharjah, ARE; 3 School of Medicine, Gulf Medical University, Ajman, ARE; 4 Hepato-Pancreato-Biliary and Transplant Surgery, King’s College Hospital London – Dubai, Dubai, ARE

**Keywords:** biliary cystadenoma, cystic liver lesion, hepatic cystic tumor, hepatobiliary surgery, liver resection, multiloculated hepatic cyst, steatohepatitis

## Abstract

Biliary cystadenomas are benign cystic neoplasms of the liver with recognized malignant potential. They are frequently asymptomatic and are often discovered incidentally during imaging for unrelated complaints. Early identification and complete surgical excision are recommended to prevent malignant transformation. We report the case of a 47-year-old male who was incidentally found to have a large, complex hepatic cyst during evaluation for mild, non-specific abdominal discomfort. Cross-sectional imaging revealed an 8 × 9.4 × 9 cm multiloculated cystic lesion involving hepatic segments VII and VIII, without radiological features suggestive of malignancy. The patient underwent elective open anatomical resection of segments VII and VIII. Histopathological examination confirmed a benign biliary-type cystadenoma, with background liver parenchyma showing severe steatohepatitis involving more than 60% of hepatocytes. The postoperative course was uneventful. This case highlights the importance of definitive surgical management of large biliary cystadenomas, even in asymptomatic patients, and underscores the added clinical considerations when significant hepatic steatosis is present.

## Introduction

Biliary cystadenomas are benign cystic tumors of the liver, accounting for less than 5% of all hepatic cystic lesions [1]. Although histologically benign, these tumors are considered premalignant due to the potential for transformation into biliary cystadenocarcinoma. For this reason, complete surgical excision is recommended once the diagnosis is suspected. Imaging and clinical presentation are often nonspecific, and these lesions are frequently discovered incidentally due to slow growth and lack of early symptoms [1]. Multiloculated architecture and internal septations may suggest the diagnosis, but histopathological confirmation remains essential. Biliary cystadenomas most commonly occur in middle-aged women, possibly due to hormonal influences and ovarian-type stroma in some lesions [2]. However, cases in male patients are documented and may contribute to diagnostic delay or misclassification [2]. Extrahepatic and intrahepatic variants of biliary cystic neoplasms further emphasize diagnostic complexity [3]. Because of these factors, surgical series and outcome studies consistently support definitive resection as the optimal treatment for preventing recurrence and malignant progression [4,5]. This report describes a case of biliary cystadenoma in a male patient with coexisting severe steatohepatitis. The case underscores diagnostic evaluation, surgical decision-making, and management of concurrent liver disease to optimize postoperative outcomes.

## Case presentation

**Figure 1:**
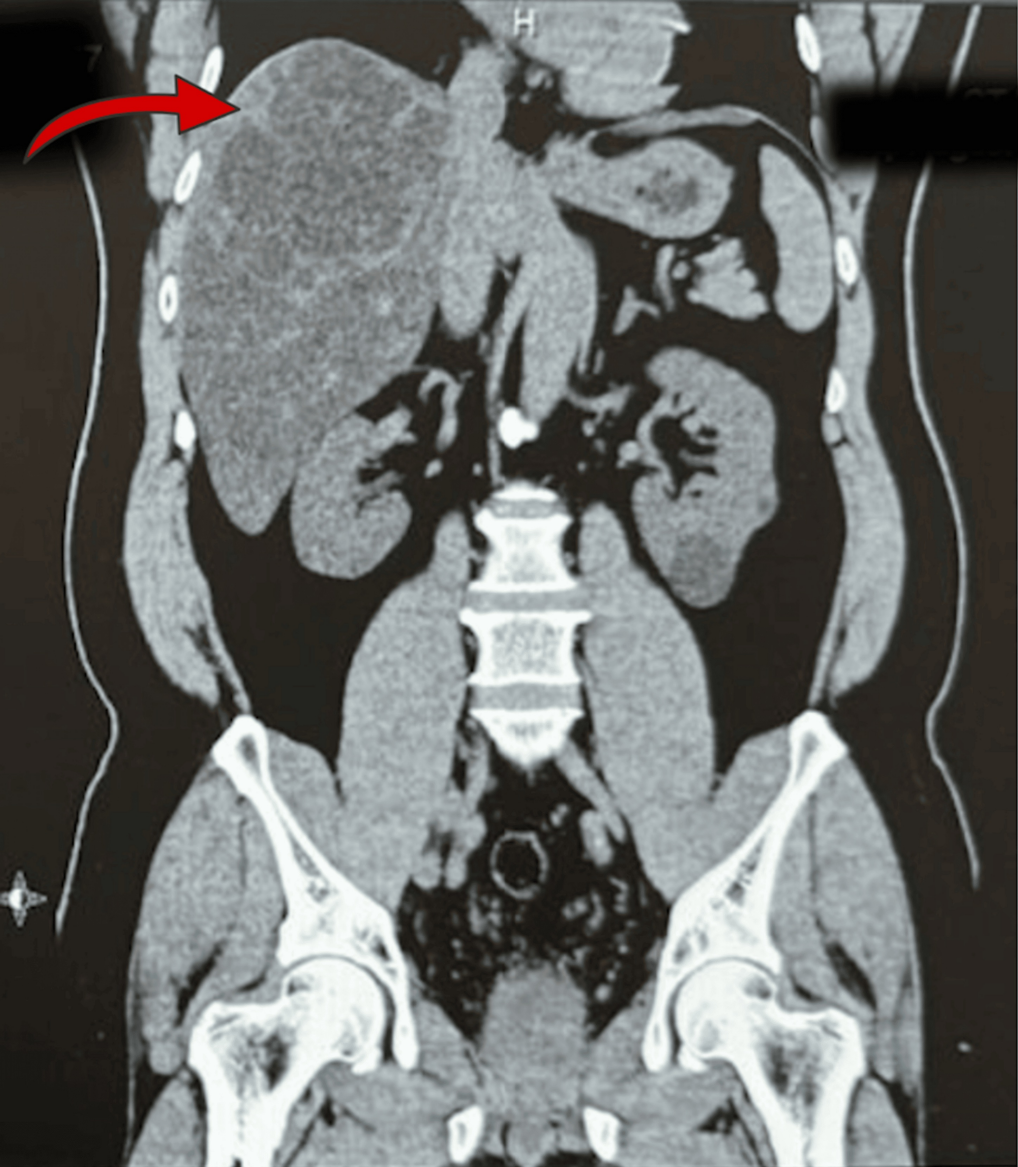
Computed tomography (CT) of the abdomen (coronal view) Demonstrating a large, well-defined multiloculated (hypodense) cystic lesion within the right hepatic lobe, with associated hepatic steatosis and posterior displacement of the inferior vena cava.

**Figure 2:**
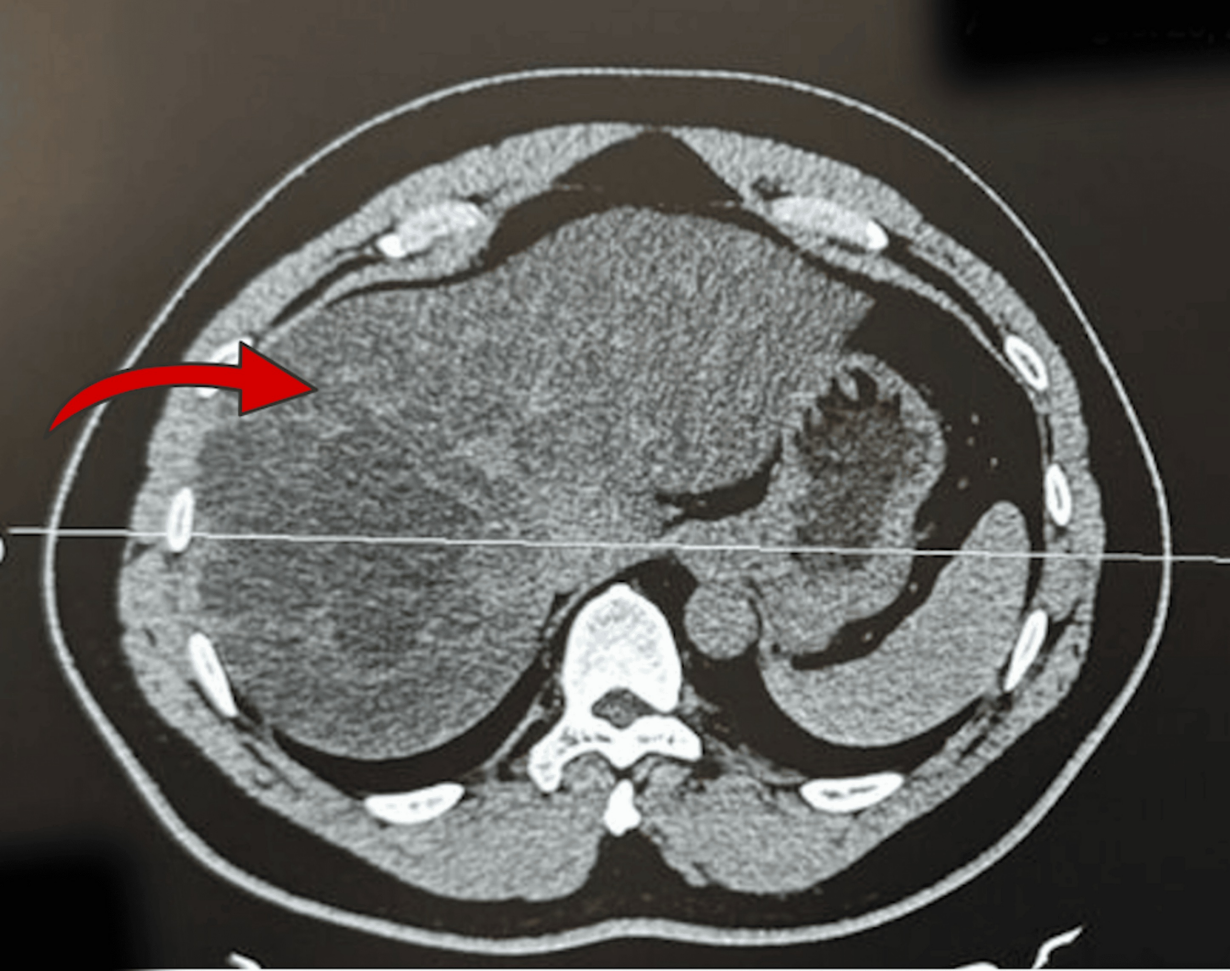
Computed tomography (CT) of the abdomen (axial view) Axial CT image showing the cystic lesion involving segments VII and VIII of the liver, without enhancing solid components.

**Figure 3:**
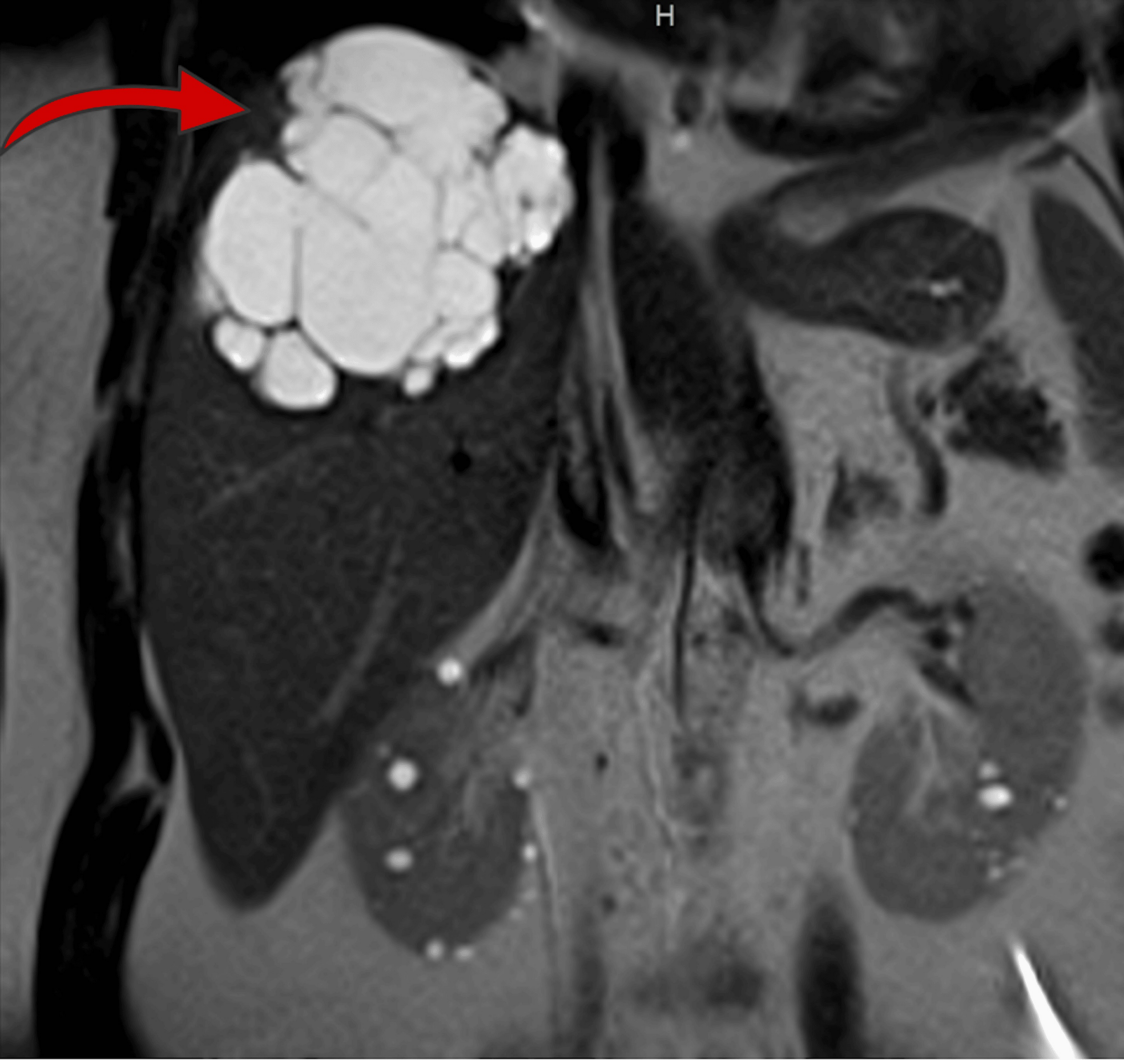
Magnetic resonance imaging (MRI) of the abdomen (coronal view) Coronal MRI image showing multiloculated cystic lesion measuring 8 × 9.4 × 9 cm involving hepatic segments VII and VIII

A 47-year-old male, with a history of hypertension and hyperlipidemia (both well controlled with medication), was referred following incidental detection of a large hepatic cyst during ultrasound evaluation for mild, non-specific abdominal discomfort. He denied abdominal pain, jaundice, fever, weight loss, or other systemic symptoms. Physical examination revealed a soft, non-tender abdomen without palpable masses or hepatosplenomegaly. On admission, vital signs were stable: temperature 36.6 °C, heart rate 64 beats per minute, respiratory rate 18 breaths per minute, blood pressure 118/67 mmHg, and oxygen saturation 98% on room air. Baseline laboratory investigations, including complete blood count, coagulation profile, liver function tests, and metabolic parameters, were within normal limits except for mild elevation of alanine aminotransferase and random glucose. The full laboratory data and reference ranges are summarized in Table 1. Contrast-enhanced computed tomography (CT) demonstrated a well-defined multiloculated cystic lesion measuring 8 × 9.4 × 9 cm involving hepatic segments VII and VIII, without enhancing mural nodules or solid components (Figures 1, 2). Mild compression of the inferior vena cava and displacement of adjacent hepatic veins were noted. Magnetic resonance imaging (MRI) further confirmed the multiloculated cystic morphology without suspicious enhancement (Figure 3). Given the lesion’s size, complex morphology, and proximity to major vascular structures, surgical resection was recommended.

**Table 1:** Routine laboratory investigations and vital signs on admission WBC: white blood cell count; RBC: red blood cell count; PT: prothrombin time; INR: international normalized ratio; PTT: partial thromboplastin time; SpO₂: peripheral oxygen saturation; MCV: mean corpuscular volume; MCH: mean corpuscular hemoglobin; MCHC: mean corpuscular hemoglobin concentration; RDW: red cell distribution width; P-LCR: platelet large cell ratio; PCT: plateletcrit; ALP: alkaline phosphatase; ALT: alanine aminotransferase; AST: aspartate aminotransferase; BUN: blood urea nitrogen; eGFR: estimated glomerular filtration rate; CRP: C-reactive protein

Category	Parameter	Result	Units	Reference range
Vital Signs	Temperature	36.6	°C	36.1–37.2 °C
	Heart rate	64	beats per minute	60–100 beats per minute
	Respiratory rate	18	breaths per minute	12–20 breaths per minute
	Blood pressure	118/67	mmHg	<120/80 mmHg
	Oxygen saturation (SpO2)	98	% (room air)	≥95%
Coagulation	Prothrombin time (PT)	12.6	seconds	11–13.5 seconds
	International normalized ratio (INR)	0.95	ratio	0.8–1.2
	Partial thromboplastin time (PTT)	35.1	seconds	25–35 seconds
Blood Group	ABO type	O		
	Rhesus (Rh) factor	Positive		
	Antibody screen	Negative		
CBC	White blood cell count (WBC)	6.8	x10^3^/µL	4.0–11.0 x10^3/µL
	Red blood cell count (RBC)	5.43	x10^6^/µL	4.5–5.9 x10^6/µL
	Hemoglobin	16.6	g/dL	13.5–17.5 g/dL
	Hematocrit	48	%	41–53%
	Mean corpuscular volume (MCV)	88	fL	80–100 fL
	Mean corpuscular hemoglobin (MCH)	31	pg	27–33 pg
	Mean corpuscular hemoglobin concentration (MCHC)	35	g/dL	32–36 g/dL
	Red cell distribution width (RDW)	12.9	%	11.5–14.5%
	Platelet count	334	x10^3^/µL	150–400 x10^3/µL
	Platelet large cell ratio (P-LCR)	13	%	13–43%
	Plateletcrit (PCT)	0	%	0.1–0.5%
CBC Differential (%)	Neutrophils	58	%	40–70%
	Lymphocytes	31	%	20–45%
	Monocytes	8	%	2–10%
	Eosinophils	2	%	1–6%
	Basophils	0.4	%	0–1%
	Nucleated RBC	0	%	0%
CBC Differential (Absolute)	Neutrophils absolute	3.97	x10^3^/µL	1.5–7.5 x10^3/µL
	Lymphocytes absolute	2.1	x10^3^/µL	1.0–4.0 x10^3/µL
	Monocytes absolute	0.57	x10^3^/µL	0.2–0.8 x10^3/µL
	Eosinophils absolute	0.1	x10^3^/µL	0.0–0.5 x10^3/µL
	Basophils absolute	0.03	x10^3^/µL	0.0–0.2 x10^3/µL
	Nucleated RBC absolute	0	x10^3^/µL	0 x10^3/µL
Liver Function	Total potein	7.4	g/dL	6.0–8.3 g/dL
	Albumin	4.7	g/dL	3.5–5.0 g/dL
	Globulin	2.7	g/dL	2.0–3.5 g/dL
	Albumin/globulin ratio	1.7	ratio	1.0–2.2
	Alkaline phosphatase (ALP)	94	U/L	44–147 U/L
	Alanine aminotransferase (ALT)	100	U/L	7–56 U/L
	Aspartate aminotransferase (AST)	42	U/L	10–40 U/L
	Total bilirubin	0.48	mg/dL	0.1–1.2 mg/dL
	Direct bilirubin	0.18	mg/dL	0.0–0.3 mg/dL
	Indirect bilirubin	0.3	mg/dL	0.2–0.8 mg/dL
Renal/Metabolic	Blood urea nitrogen (BUN)	24	mg/dL	7–20 mg/dL
	Serum urea	6.43	mmol/L	2.5–7.8 mmol/L
	Creatinine	1.19	mg/dL	0.6–1.3 mg/dL
	Estimated glomerular filtration rate (eGFR)	85	mL/min/1.73m²	≥90 mL/min/1.73m²
	BUN/creatinine ratio	20	ratio	10–20
	Sodium	140	mmol/L	135–145 mmol/L
	Potassium	4.2	mmol/L	3.5–5.0 mmol/L
	Chloride	106	mmol/L	98–106 mmol/L
	Bicarbonate	28	mmol/L	22–29 mmol/L
	Calcium	9.1	mg/dL	8.6–10.2 mg/dL
	Phosphorus	2.8	mg/dL	2.5–4.5 mg/dL
	Magnesium	2	mg/dL	1.7–2.2 mg/dL
	Random glucose	182	mg/dL	<140 mg/dL (non-fasting)
Cardiac/Inflammatory	Troponin I	<0.10	ng/mL	<0.04 ng/mL
	C-reactive protein (CRP)	1	mg/L	<5 mg/L

Surgical management and histopathology

**Figure 4:**
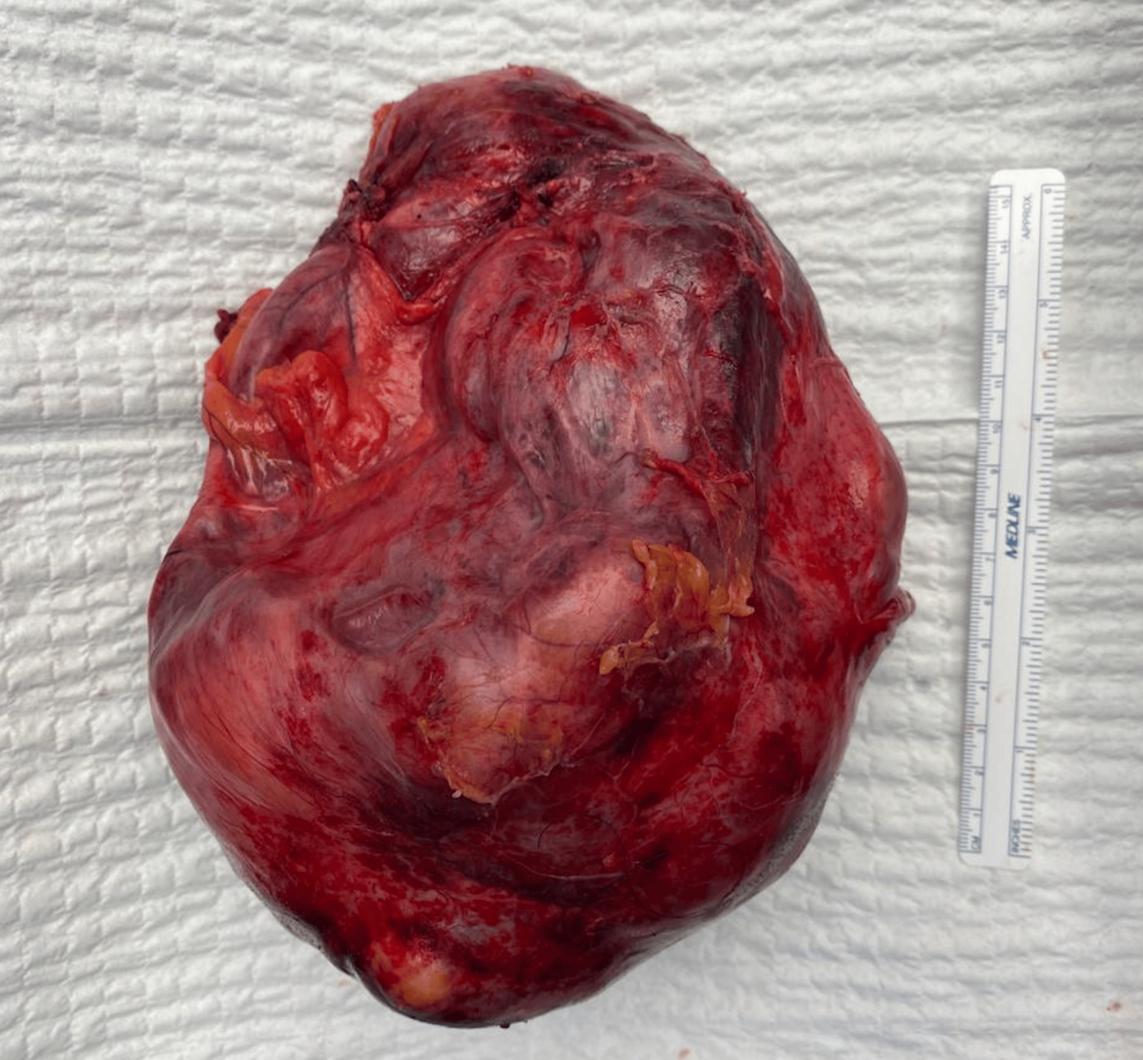
Resected specimen Large, well-encapsulated multiloculated cystic hepatic mass with smooth external surface and areas of congestion, consistent with biliary cystadenoma.

The patient underwent elective open anatomical resection of hepatic segments VII and VIII under general anesthesia with epidural analgesia. Following mobilization of the right hepatic lobe, intraoperative ultrasound confirmed the cyst’s close relationship to the hepatic veins and IVC. Parenchymal transection was performed using ultrasonic dissection, with intermittent portal triad clamping for a total of 15 minutes. The resected specimen was a large, well-encapsulated multiloculated cystic mass with a smooth external surface (Figure 4). Estimated blood loss was approximately 1500 mL, and no intraoperative complications occurred. Histopathological examination revealed a multiloculated cyst lined by biliary-type epithelium, positive for cytokeratin 7 (CK7), with no evidence of dysplasia or malignancy, confirming the diagnosis of biliary cystadenoma. Background liver parenchyma demonstrated severe steatohepatitis, involving more than 60% of hepatocytes. The postoperative course was uneventful. The patient achieved complete wound healing by postoperative day 12 and reported minimal pain. He was discharged with recommendations for hepatology follow-up and lifestyle modifications to address hepatic steatosis.

## Discussion

Biliary cystadenomas are rare cystic hepatic neoplasms with recognized malignant potential [1]. Although benign, these lesions may progress to cystadenocarcinoma, justifying complete surgical excision once diagnosed [3]. Imaging features such as multiloculated architecture and internal septations raise suspicion, but radiology alone cannot reliably exclude malignancy. Histopathological confirmation remains essential for diagnosis and treatment planning. Most biliary cystadenomas are discovered incidentally due to their indolent growth and nonspecific symptoms [1]. While predominantly reported in women, occurrence in male patients is documented and may contribute to delayed diagnosis [2].

Our case reflects these diagnostic challenges, with the lesion identified incidentally and initially lacking clinical manifestations. Complete surgical resection remains the gold standard treatment because conservative approaches are associated with high recurrence and potential malignant transformation [5,6,7,8]. Literature reviews and outcome studies demonstrate excellent prognosis following complete excision, whereas incomplete treatment increases recurrence risk [8]. In this case, anatomical resection achieved definitive management without complications. The coexistence of severe steatohepatitis adds clinical complexity. Hepatic steatosis can impair regenerative capacity and increase postoperative risk [4]. Comprehensive preoperative assessment and long-term metabolic management are essential to optimize outcomes and prevent future hepatic complications [8]. Similar considerations are emphasized in studies addressing surgical outcomes in patients with coexisting liver disease. This case supports existing evidence that biliary cystadenomas require aggressive surgical management to prevent malignant transformation and recurrence [5,6]. Multidisciplinary evaluation and meticulous surgical planning are essential for optimal results.

## Conclusions

Biliary cystadenomas should be considered in the differential diagnosis of complex hepatic cysts, regardless of patient gender or symptom burden. Early recognition and complete surgical resection are essential to prevent malignant transformation and future complications. This case further highlights the importance of addressing coexisting liver pathology, such as steatohepatitis, as part of comprehensive patient management. An integrated approach combining accurate diagnosis, definitive surgical treatment, and long-term metabolic control offers optimal outcomes for patients with complex cystic liver lesions.
